# Supplementary figures and images for: Does a smart business environment promote corporate investment? a case study of Hangzhou
Source: PLoS One. 2022 Jul 6;17(7):e0269089. doi: 10.1371/journal.pone.0269089 (PMC9258851; doi:10.1371/journal.pone.0269089)

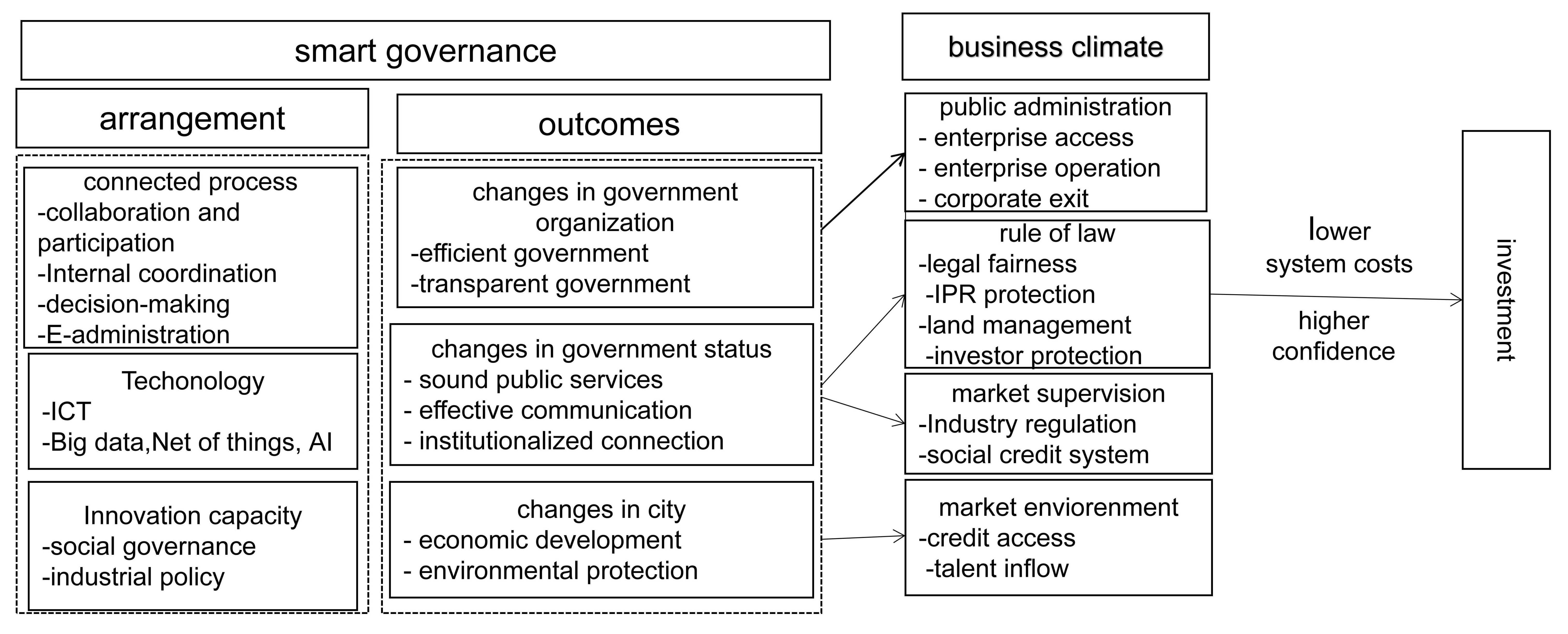

Supplement: S1 Fig — Fig 1 shows our theoretical framework. (PNG) [file pone.0269089.s001.png]

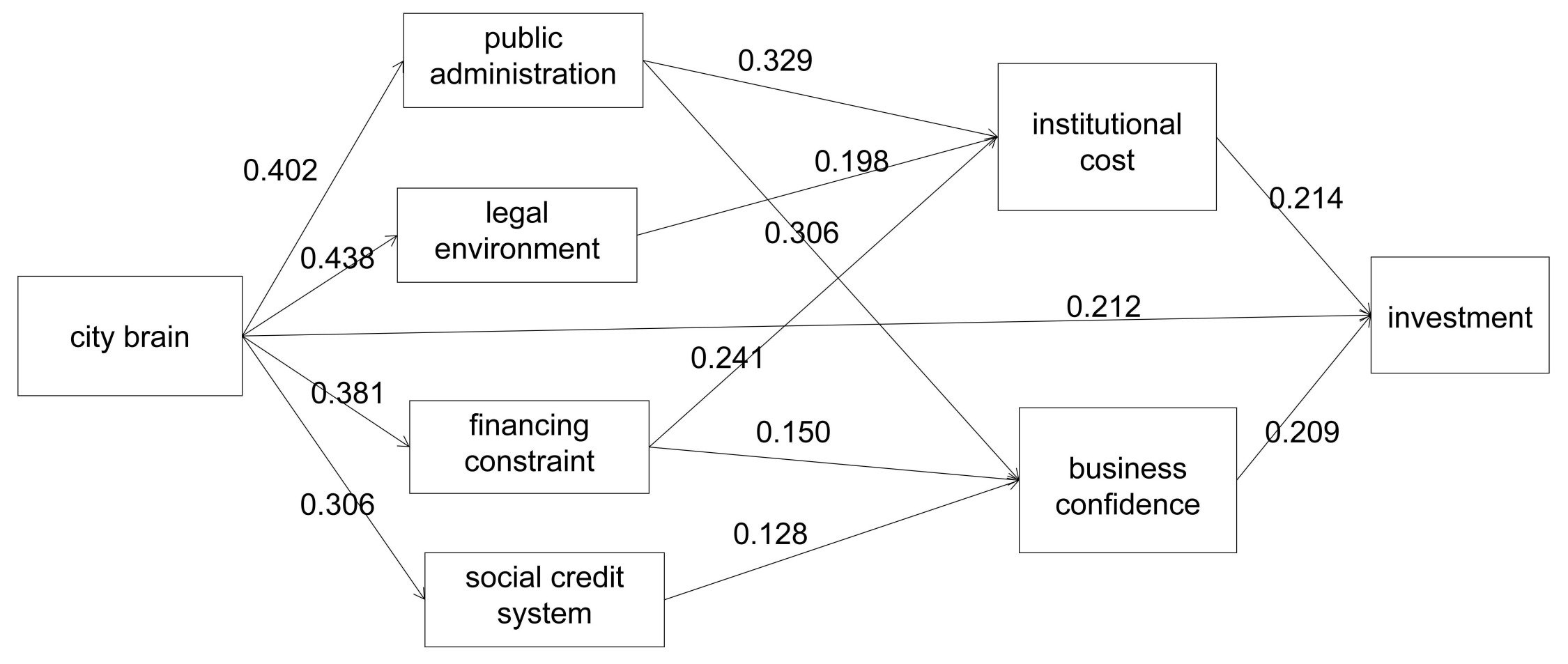

Supplement: S2 Fig — Fig 2 shows the overall influence paths among smart governance, business environments, institutional costs, business confidence, and enterprise investments. (PNG) [file pone.0269089.s002.png]
