# Supplementary material for: Does a smart business environment promote corporate investment? a case study of Hangzhou
Source: PLoS One. 2022 Jul 6;17(7):e0269089. doi: 10.1371/journal.pone.0269089 (PMC9258851; doi:10.1371/journal.pone.0269089)
Supplement: Attachment — Submitted filename: Response_letter_20220326.docx [file pone.0269089.s003.docx]

Response letter

Emily Chenette

Editor-in-Chief

*PLOS ONE*

Dear Editor:

I, along with my co-authors, would like to re-submit the attached manuscript entitled “Does a smart business environment promote corporate investment? A case study of Hangzhou.” The manuscript ID is PONE-D-21-22763.

Thank you for giving me the opportunity to submit the revised version of my manuscript. I appreciate the time and effort that you have dedicated to providing feedback on my manuscript and I am grateful for the insightful comments, which allowed me to make valuable improvements to the paper.

I have incorporated most of the suggestions made by you and the reviewers. Below, please find the point-by-point responses to the comments and concerns.

I hope these changes fully address the reviewers’ concerns and make the paper suitable for publication in your journal.

Thank you for your consideration. I look forward to hearing from you.

Sincerely,

H.-Y. Song

Zhejiang International Studies University

Email: 5374514@qq.com

Reviewer #1:

Comment 1

1. The manuscript is not technically sound, and the data doesn’t support the conclusions.

Response:

Thank you for the comment. We are sorry but we do not agree with your opinion.

We examined the impact of a smart local business environment on the corporate investment scale in Hangzhou, China, using survey data from 297 firm managers. We use factor analysis and linear regression to verify Propositions 2-4, and adopt path analyses to prove Proposition 1.

Four hypotheses are tested. First, smart governance can improve the business environment. This proposition has not been fully addressed in the literature. Second, smart environments for government services, financing, and the rule of law can enhance enterprise confidence and reduce institutional costs. Third, enhanced business confidence and lower institutional costs lead to a larger investment scale. The relationship between commercial climate, institutional costs, and business confidence has long been observed and analyzed, but the impact of a smart business environment on institutional costs and business confidence has not been fully explored.

Comment 2

The statistical analysis hasn’t been performed appropriately and rigorously.

Response:

Thank you for the comment. We are sorry, but we do not agree with your opinion.

First of all, we designed a questionnaire based on a large amount of domestic and foreign literature.

Second, we strictly followed the scientific procedure of random sampling to ensure the randomness of sampling.

Third, we strictly tested the questionnaire data to ensure the reliability and validity of the questionnaire results.

Fourth, we designed the empirical estimation model based on the scientific theoretical framework.

Fifth, we carefully explained the estimated results of the model and compared them with the conclusions of a large number of relevant studies in China and abroad to enhance the credibility of our research results.

Comment 3

The authors haven’t made all data underlying the findings in their manuscript fully available.

Response:

Thank you for the comment. This time we supply all the survey data as supporting materials.

The revisions are in supporting materials.

Comment 4

the manuscript isn’t presented in an intelligible fashion and written in standard English.

Response:

Thank you for the comment. The manuscript has been proofread again by the professional editing agency, Editage.

Comment 5

The work is useful but the paper lacks a clear structure.

Response:

Thank you for the comment. We have carried out the following revision to make the structure clearer.

First, we integrate the discussion of linear regression into the results of linear regression. Second, the format is revised according to the journal’s requirements. Third, 22 tables have been revised to only 11 tables to make the structure of the article clearer.

Comment 6

The textual and graphical presentation is poor.

Response:

Thank you for the comment. We have reduced 22 tables to 11 tables and redrawn the 2 figures at a higher resolution. In addition, the manuscript has undergone proofreading by the professional editing agency, Editage.

Comment 7

Some equations are not properly typed.

Response:

Thank you for the comment. We have revised the equations according to the journal’s requirement.

Reviewer #2:

Comment 1

The manuscript is technically sound, and the data supports the conclusions.

The statistical analysis has been performed appropriately and rigorously.

The manuscript is presented in an intelligible fashion and written in standard English.

Response:

Thank you very much for your appreciation

Comment 2

The authors haven’t made all data underlying the findings in their manuscript fully available.

Response:

Thank you for the comment. This time we supply all the survey data as supporting materials.

Comment 3

The authors have made a good effort to combine business environment and smart city concepts. It is a good paper and can be accepted subjected to major modifications.

Response:

Thank you for the comment. We have improved the scope, implications, contributions, and presentation.

Comment 4

It would be helpful if further analysis included other variables such as infrastructure and labor quality, which are important factors.

Response:

Thank you for your advice. We agree with your opinion.

Our aim is examining the impact of smart governance on enterprise investment; therefore, we have added B2B intermediary services and entrepreneurship supporting facilities as indicators of urban infrastructure. We have also added talent (qualified numbers of talents) into our regression. The results show that infrastructure promotes business confidence, and talent decreases operational costs but increases entry costs. This result implies talent shortages hinder the beneficial functions of a smarter business environment.

Comment 5

The case study is limited as it focuses on one Chinese city in 2018. Although the authors acknowledge that their study is limited to a small number of developing countries, they do not discuss whether the results can be generalized to other developing countries. (line 101)

Response:

Thank you for the comment. We agree with you and have added the discussion of generalization of the results as follows.

The results can be generalized to other developing countries. First, our conclusion on the limitation effect of intelligent business environment on enterprise investment can inspire other scholars to conduct further research on the intersection of business environments and smart cities. Second, the rules of an intelligent business environment in promoting corporate investment are still in line with the reality of other countries. As the largest developing country in the world, China's economic development is particularly diversified. The law of intelligent business environment attracting investment, obtained in China’s context, is of great significance for other developing countries. Especially now, when the world economic environment is greatly impacted by uncertainty, countries can attract investment and promote economic development through intelligent governance.

Comment 6

The study did not include any discussion of climate change or environmental factors which are increasingly becoming a concern for business decision-makers around the world.

Response:

Thank you for the comments. Our study lasted only for one year and was a cross-sectional study; therefore, it was difficult to reflect the impact of intelligent governance on the improvement of natural environment or climate change. However, that does not mean that it has no such effect. We will focus on this topic in our future research.

Comment 7

Another concern is the quality of the figures and tabular results. I suggest the authors improve the presentation of the tabular results and consider presenting these using figures/plots. The figure quality should be improved.

Response:

Thank you for the comment. We have reduced 22 tables to 11 tables and redrawn the 2 figures at a higher resolution. In addition, the manuscript has been proofread again by the professional editing agency, Editage.

Comment 8

Page-17: lines 363-369 some missing formula characters.

Response:

Thank you for the comment. We have added the formula characters.
